# Therapeutic Effects of Inhibitor of *ompA* Expression against Carbapenem-Resistant *Acinetobacter baumannii* Strains

**DOI:** 10.3390/ijms222212257

**Published:** 2021-11-12

**Authors:** Seok-Hyeon Na, Hyejin Jeon, Man-Hwan Oh, Yoo-Jeong Kim, Mingi Chu, Ill-Young Lee, Je-Chul Lee

**Affiliations:** 1 Division of Antimicrobial Resistance Research, Center for Infectious Diseases Research, National Institute of Infectious Diseases, National Institute of Health, Korea Disease Control and Prevention Agency, Cheongju 28159, Korea; nash8090@korea.kr; 2 Department of Microbiology, School of Medicine, Kyungpook National University, Daegu 41944, Korea; hyejin7432@knu.ac.kr (H.J.); gracekim023@naver.com (Y.-J.K.); 3 Department of Microbiology, College of Science and Technology, Dankook University, Cheonan 16890, Korea; yy1091@dankook.ac.kr; 4 Research Center for Eco-Friendly New Materials, Bio & Drug Discovery Division, Korea Research Institute of Chemical Technology, Daejeon 34114, Korea; mgchu@naver.com (M.C.); iylee@krict.re.kr (I.-Y.L.)

**Keywords:** *Acinetobacter baumannii*, *ompA* promoter inhibitor, compound 62520, anti-virulence, bacteriostatic agent

## Abstract

The widespread of carbapenem-resistant *Acinetobacter baumannii* (CRAB) is of great concern in clinical settings worldwide. It is urgent to develop new therapeutic agents against this pathogen. This study aimed to evaluate the therapeutic potentials of compound 62520, which has been previously identified as an inhibitor of the *ompA* promoter activity of *A. baumannii*, against CRAB isolates, both in vitro and in vivo. Compound 62520 was found to inhibit the *ompA* expression and biofilm formation in *A. baumannii* ATCC 17978 at sub-inhibitory concentrations in a dose-dependent manner. These inhibitory properties were also observed in clinical CRAB isolates belonging to sequence type (ST) 191. Additionally, compound 62520 exhibited a bacteriostatic activity against clinical clonal complex (CC) 208 CRAB isolates, including ST191, and ESKAPE pathogens. This bacteriostatic activity was not different between STs of CRAB isolates. Bacterial clearance was observed in mice infected with bioimaging *A. baumannii* strain 24 h after treatment with compound 62520. Compound 62520 was shown to significantly increase the survival rates of both immunocompetent and neutropenic mice infected with *A. baumannii* ATCC 17978. This compound also increased the survival rates of mice infected with clinical CRAB isolate. These results suggest that compound 62520 is a promising scaffold to develop a novel therapeutic agent against CRAB infections.

## 1. Introduction

*Acinetobacter baumannii* is one of the problematic ‘ESKAPE’ pathogens, known to be the leading cause of nosocomial infections and to develop multidrug resistance (MDR) [1]. This gram-negative, non-fermenting opportunistic pathogen causes a variety of nosocomial infections—including pneumonia, wound and urinary tract infections, meningitis, and bacteremia—in severely ill patients admitted to intensive care units [2,3,4]. *A. baumannii* has developed resistance to the commonly used antimicrobial agents through both intrinsic resistance and the acquisition of resistant determinants [3,4]. Carbapenems have been widely used to treat MDR *A. baumannii* infections during the last two decades, resulting in the prevalence of carbapenem-resistant *A. baumannii* (CRAB) in many countries [5,6,7]. Colistin and tigecycline still work effectively for the treatment of CRAB infections, but resistance to these antimicrobial agents has also increased in clinical *A. baumannii* isolates [8,9]. The wide spread of drug-resistant *A. baumannii* strains, and the therapeutic difficulty associated with it, are of great concern in clinical settings worldwide [10,11]. In light of the gravity of such antimicrobial resistance, it is urgent to develop new therapeutic agents against drug-resistant *A. baumannii*.

The implementation of anti-virulence strategies to target and control virulence factors is one of the potential alternative approaches to combat drug-resistant pathogens [12,13]. However, the development of anti-virulence agents against *A. baumannii* is still in its early stages. In this regard, studies of *A. baumannii* have shown that the small organic molecule virstatin inhibits biofilm formation and surface motility in vitro [14], and the flavonoid curcumin performs the same functions, while also inhibiting pellicle formation in this bacterium [15]. Similarly, the natural, plant-derived chemical compound zerumbone was shown to inhibit biofilm formation, and also to disrupt established biofilms [16]. Other studies indicated that a synthetic cyclic peptide, known as AOA-2, and its derivatives, bind to the outer membrane protein A (OmpA) of *A. baumannii*, and reduce bacterial virulence both in vitro and in vivo [17,18]. This synthetic peptide increases the colistin susceptibility against colistin-resistant *A. baumannii* [19]. The OmpA is considered as a new therapeutic target for anti-virulence agents against *A. baumannii* [20], as it is not only responsible for the structural rigidity and permeability of small solutes, but also plays a role in the virulence and antimicrobial resistance of this pathogen [20,21,22,23]. The Δ*ompA* mutant is less virulent and more susceptible to antimicrobial agents than the wild-type (WT) *A. baumannii* strain is [23,24]. OmpA production is tightly regulated by post-transcriptional stages in *Escherichia coli* [25,26,27]. In addition, we recently demonstrated the transcriptional regulation of *ompA* in *A. baumannii* [28], suggesting that the regulation of *ompA* expression can control the OmpA-mediated virulence of *A. baumannii*. In this context, we previously screened small molecules that inhibit the *ompA* promoter activity using the reporter strain *A. baumannii* ATCC 17978 carrying the plasmids containing the *ompA* promoter and *npt*I conferring resistance to kanamycin [29]. Of the identified small molecules inhibiting the *ompA* promoter activity, it was found that compound 62520 (5-fluoro-1-((1R,3S)-3-(hyroxymethyl)-1.3-dihydroisobenzofuran-1-yl)pyrimidine-2,4-(1H,3H)-dione) down-regulated the expression of *ompA* and OmpA in the outer membrane, and attenuated biofilm formation in *A. baumannii* ATCC 17978 at 1.0 μM. However, this compound showed bacteriostatic activity against *A. baumannii* ATCC 17978 at ≥20 μM. These results suggest that compound 62520 has not only an anti-virulence activity, such as the OmpA-mediated anti-biofilm activity, at sub-inhibitory concentrations, but also an antimicrobial activity at relatively high concentrations. Therefore, in this study, we evaluated the OmpA-mediated anti-virulence and antimicrobial activities of compound 62520 against CRAB strains, both in vitro and in vivo.

## 2. Results

### 2.1. Compound 62520 Is a Hit Small Molecule Inhibiting the ompA Promoter Activity of A. baumannii

Reporter strain OH101, carrying a single copy of the *ompA* promoter and an open reading frame of *ermAM* fusion in the chromosome, was constructed to determine whether compound 62520 inhibited the *ompA* promoter located in the chromosome. This compound exhibited a 72.2% growth inhibition of reporter strain OH101 at 2.5 μM (0.7 μg/mL) (Supplementary Table S1). The inhibitory activity of compound 62520 in reporter strain OH101 under erythromycin conditions (30 μg/mL) was similar to that of reporter strain OH102 (72.0%) under kanamycin conditions (50 μg/mL) [29]. Next, 95 chemical compounds that showed structural similarity with compound 62520 were selected from the chemical library of the Korea Chemical Bank (http://www.chembank.org/, accessed on 1 February 2018), and their ability to inhibit the growth of the OH101 strain in a culture medium containing erythromycin was then determined. Fourteen chemical compounds with this inhibitory effect were identified (Supplementary Table S1), and compound 62520 was the most active of all in inhibiting the growth of both reporter strains OH101 and OH102. Finally, this compound was selected as a hit small molecule, and its anti-OmpA and antimicrobial properties against *A. baumannii* strains in vitro and in vivo were further tested.

### 2.2. Compound 62520 Inhibits ompA Expression and Biofilm Formation in A. baumannii ATCC 17978 and CRAB Isolates at Sub-Inhibitory Concentrations

**Figure 1:**
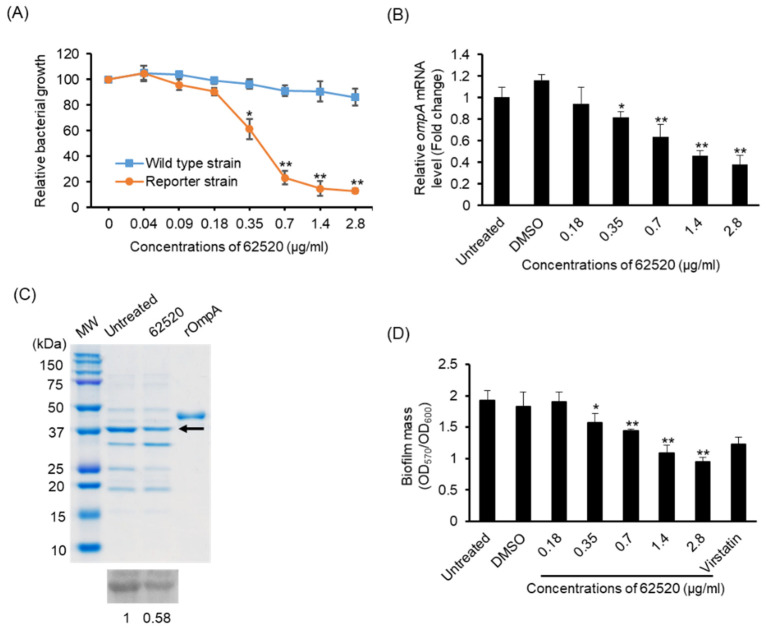
Activity of compound 62520 at sub-inhibitory concentrations. (**A**) The WT *A. baumannii* ATCC 17978 and reporter strain OH101 were treated with different concentrations of compound 62520 for 24 h and bacterial growth was measured at OD_600_. The relative bacterial growth was determined by the OD_600_ in each concentration of compound 62520 relative to the OD_600_ in the control (0 μg/mL). Data are presented as the mean ± SD of three independent experiments. (**B**) *A. baumannii* ATCC 17978 was treated with different concentrations of compound 62520 for 24 h and then total RNA was extracted. The expression levels of *ompA* were analyzed by qPCR. Data are presented as the mean ± SD of the expression levels of *ompA* in *A. baumannii* ATCC 17978 treated with compound 62520 relative to those of untreated control bacteria. Data were obtained from three independent experiments. (**C**) SDS-PAGE and western blotting of the outer membrane fractions in *A. baumannii* ATCC 17978. The arrow indicates the OmpA protein. The values in western blotting represent the intensity of bands relative to the intensity of untreated bacteria. (**D**) *A. baumannii* ATCC 17978 was inoculated in 96-well plates and different concentrations of compound 62520 were then added. Bacteria were treated with virstatin (100 μM) as a positive control. The biofilm mass was quantitated as the OD_570_ normalized to the OD_600_. Data are presented as the mean ± SD of three independent experiments. * *p* < 0.05, ** *p* < 0.01 compared with the control bacteria.

The dose-responsiveness of compound 62520 was determined in the growth of reporter strain OH101 and WT *A. baumannii* ATCC 17978. Compound 62520 was shown to significantly inhibit the growth of reporter strain OH101 at concentrations ≥0.35 μg/mL in a dose-dependent manner, but it did not significantly inhibit the growth of the WT strain at concentrations ≤2.8 μg/mL (Figure 1A). To determine whether it inhibited *ompA* expression in *A. baumannii* ATCC 17978, bacteria were treated with different concentrations of the compound for 24 h, and then *ompA* expression was analyzed using quantitative real-time polymerase chain reaction (qPCR). The expression of this gene was significantly inhibited at ≥0.35 μg/mL in a dose-dependent manner (Figure 1B) and, along with this inhibition, compound 62520 also inhibited the expression of OmpA in the outer membrane of *A. baumannii* ATCC 17978 at 0.7 μg/mL (Figure 1C). In addition, it inhibited biofilm formation in the same bacterium in a dose-dependent manner (Figure 1D). Specifically, its inhibitory effect at 2.8 μg/mL (10 μM) on biofilm formation was more active than that of virstatin (100 μM).

**Figure 2:**
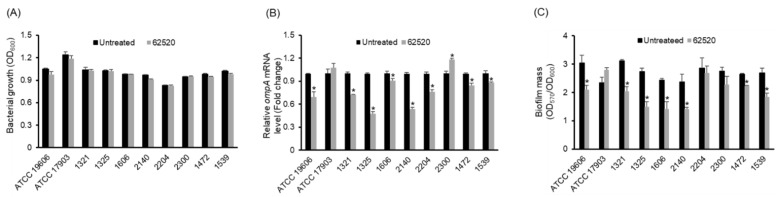
In Vitro activity of compound 62520 against *A. baumannii* ATCC 19606, *A. nosocomialis* ATCC 17903, and clinical CRAB isolates. (**A**) Bacteria were cultured with or without 2.8 μg/mL of compound 62520 for 24 h and bacterial growth was measured at OD_600_. Data are presented as the mean ± SD of three independent experiments. (**B**) Bacteria were cultured with or without 2.8 μg/mL of compound 62520 for 24 h and then total RNA was extracted. The expression levels of *ompA* were analyzed by qPCR. Data are presented as the mean ± SD of the expression levels of *ompA* in compound 62520-treated bacteria relative to those of untreated bacteria. (**C**) Bacteria were cultured with or without 2.8 μg/mL of compound 62520 for 24 h. The biofilm mass was quantitated as the OD_570_ normalized to OD_600_. For both (**B**,**C**), data are presented as the mean ± SD of three independent experiments. * *p* < 0.05 compared with the untreated bacteria.

Next, to determine whether compound 62520 inhibited *ompA* expression and biofilm formation in drug-resistant *A. baumannii* strains, eight clinical CRAB isolates belonging to sequence type (ST) 191—corresponding to global clone 2 that is the most prevalent clone worldwide—were treated with 2.8 μg/mL of the compound for 24 h, and the expression of *ompA* and biofilm formation were then analyzed. Two type strains, *A. baumannii* ATCC 19606 and *A. nosocomialis* ATCC 17903, were included. Bacterial growth was not significantly different between the control and compound 62520-treated bacteria (Figure 2A). Compound 62520 significantly inhibited *ompA* expression in *A. baumannii* ATCC 19606 and seven CRAB isolates (Figure 2B). However, an increase in *ompA* expression was observed in the CRAB 2300 isolate treated with the compound. Compound 62520 also attenuated biofilm formation in *A. baumannii* ATCC 19606 and in six ST191 CRAB isolates that showed a reduced expression of *ompA* after the treatment (Figure 2C). Compound 62520 did not inhibit *ompA* expression and biofilm formation in *A. nosocomialis* ATCC 17903.

### 2.3. Compound 62520 Has a Bacteriostatic Activity against Acinetobacter Strains

**Figure 3:**
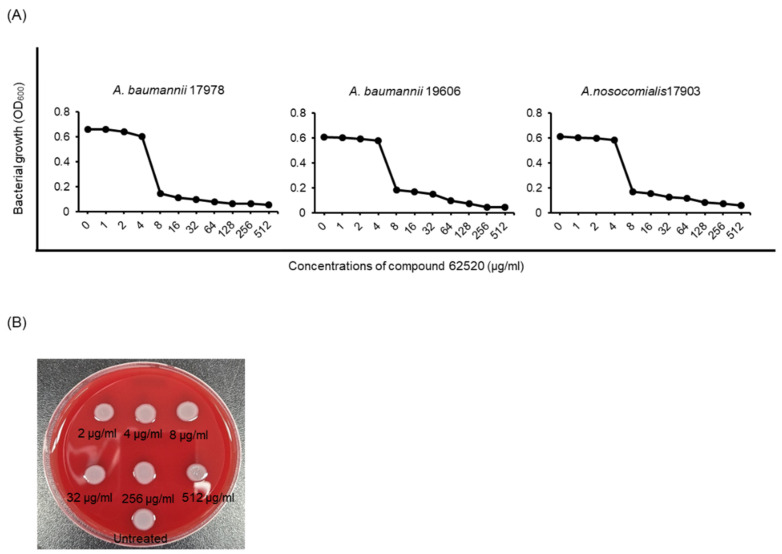
Growth inhibition of *Acinetobacter* strains by compound 62520. (**A**) Bacteria were cultured in 96-well plates and were then treated with different concentrations of compound 62520. As a control, the same volume of DMSO, used to dissolve the compound, was added (0 μg/mL). The plates were incubated for 20 h and bacterial growth was measured at OD_600_. The data are representative of two experiments that produced similar results. (**B**) *A. baumannii* ATCC 17978 treated with different concentrations of compound 62520 for 20 h was inoculated into blood agar plates and then cultured for 20 h.

Compound 62520 showed no significant antimicrobial activity against *A. baumannii* ATCC 17978 at concentrations ≤2.8 μg/mL (Figure 1A), but showed bacteriostatic activity at ≥5.6 μg/mL in a previous study [29]. To determine the dose-responsiveness of compound 62520 in the growth inhibition of *Acinetobacter* strains, two *A. baumannii* strains—ATCC 17978 and ATCC 19606—and the *A. nosocomialis* ATCC 17903 strain were incubated with the compound for 20 h. A sharp decline in the growth of all three strains was observed at a compound concentration of 8 μg/mL (Figure 3A). However, its minimum inhibitory concentrations (MICs) against the two *A. baumannii* strains were >512 μg/mL. Compound 62520 exhibited a bacteriostatic activity, but not a bactericidal one, against *A. baumannii* ATCC 17978 at ≤512 μg/mL (Figure 3B). Next, to determine whether the antibacterial activity was exhibited against clinical CRAB isolates, 25 clonal complexes (CC) 208 CRAB isolates were selected—of which five belonged to ST191, ST208, ST369, ST451, and ST784—and the MICs and half-maximal inhibitory concentration (IC_50_) of compound 62520 were determined. Nine and 16 CRAB isolates showed a sharp decline in bacterial growth at 8 μg/mL and 16 μg/mL, respectively. However, the MICs of compound 62520 against all tested CRAB isolates were >32 μg/mL (Table 1), and the mean IC_50_ ranged from 6.35 ± 3.05 μg/mL in ST784 to 8.62 ± 3.46 μg/mL in ST119. There were no significant differences in the IC_50_ of compound 62520 between STs of CRAB isolates.

### 2.4. Compound 62520 Exhibits an Antimicrobial Activity against ESKAPE Pathogens

The antimicrobial activity of compound 62520 was determined against four ESKAPE pathogens. It was revealed that the compound inhibited the growth of all tested bacteria, including *E. coli*, *Pseudomonas aeruginosa*, *Staphylococcus aureus*, and *Enterococcus faecalis*, in a dose-dependent manner (Supplementary Figure S1). Specifically, the MICs of compound 62520 against *E. coli* ATCC 25922 and *P. aeruginosa* ATCC 27853 were both >512 μg/mL. Interestingly, the MICs against *E. faecalis* ATCC 29212 and *S. aureus* ATCC 29213 were 1 μg/mL and 4 μg/mL, respectively. Compound 62520 exhibited a stronger antimicrobial effect on the gram (+) than on the gram (−) bacteria tested.

### 2.5. Compound 62520 Has a Therapeutic Effect on Mice Infected with A. baumannii Strains

**Figure 4:**
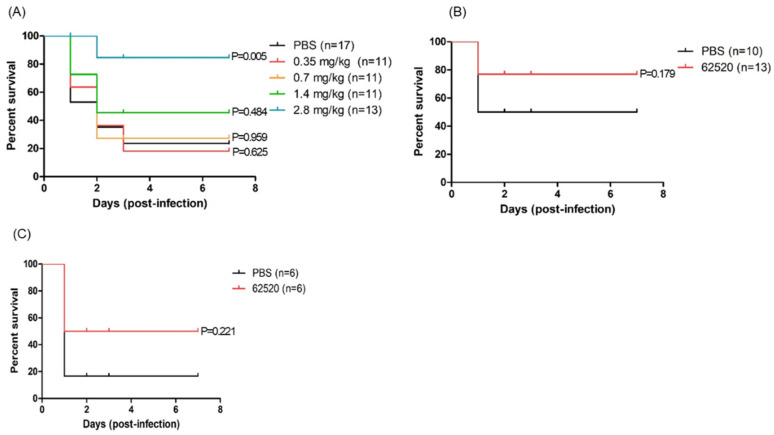
Survival rates of *A. baumannii*-infected mice treated with compound 62520. The survival of mice was evaluated for 7 days after bacterial infection. (**A**) Neutropenic mice were infected with 5 × 10^8^ CFUs of *A. baumannii* ATCC 17978 and, after 2 h, they were injected with different doses of compound 62520. (**B**) Immunocompetent mice were infected with 1.0 × 10^9^ CFUs of *A. baumannii* ATCC 17978 and, after 2 h, they were injected with 2.8 mg/kg of compound 62520. (**C**) Immunocompetent mice were infected with 2.0 × 10^8^ CFUs of *A. baumannii* 2140 and, after 2 h, they were injected with 5.6 mg/kg of compound 62520. All control mice were injected with 100 μL of PBS.

Neutropenic mice were infected with *A. baumannii* ATCC 17978 intraperitoneally and were then treated with different concentrations of compound 62520 (0.35 mg/kg to 2.8 mg/kg) to evaluate its therapeutic effects in vivo. Because *A. baumannii* commonly infects severely ill or immunocompromised patients [2,3], in this experiment, survival rates were first determined in neutropenic mice. It was found that the survival rates were significantly increased in the mice treated with 1.4 mg/kg (45.5%) and 2.8 mg/kg (84.6%) of compound 62520 compared with those treated with phosphate-buffered saline (PBS) (23.5%) (Figure 4A). However, no significant differences in the survival rates of *A. baumannii*-infected mice were observed between PBS and compound 62520 at concentrations of ≤0.7 mg/kg. A 2.8 mg/kg dosage of compound 62520 significantly increased the survival rates (76.9%) of immunocompetent mice infected with *A. baumannii* ATCC 17978 compared with those treated with PBS (50.0%) (Figure 4B). The therapeutic effects of compound 62520 against clinical CRAB isolates were assessed. Immunocompetent mice were infected intraperitoneally with 2.0 × 10^8^ colony forming units (CFUs) of ST191 CRAB isolate 2140, and compound 62520 (5.6 mg/kg) was then injected, also intraperitoneally, 2 h after bacterial injection. It was found that the compound treatment significantly increased the survival rates (50.0%) of mice compared to the PBS treatment (16.7%) (Figure 4C).

**Figure 5:**
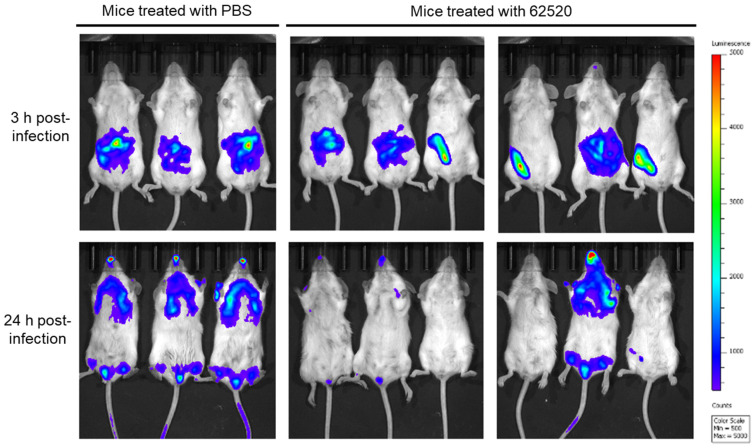
Bacterial clearance in mice treated with compound 62520. Immunocompetent mice were intraperitoneally infected with 1.0 × 10^9^ CFUs of *A. baumannii* ATCC 17978-lux strain, and, 2 h after bacterial injection, 2.8 mg/kg of compound 62520 was injected in the opposite side of their abdomen. The control mice were injected with the same volume of PBS. The color bar beside IVIS images indicates the intensity of radiance (p/sec/cm^2^/sr).

To determine whether compound 62520 had an ability to clear bacteria *in vivo*, immunocompetent mice were intraperitoneally infected with bioimaging reporter strain OH923 [24], and compound 62520 (2.8 mg/kg) was then injected intraperitoneally 2 h after bacterial injection. Then, the analysis of bacterial distribution was conducted after 3 h, and it was repeated 24 h after the infection using the in vivo imaging system (IVIS). In mice treated with PBS, bioluminescent signals were detected around the bacterial injection site at 3 h post-infection, and they were then observed in the lungs, lower abdomen, and nostrils at 24 h post-infection (Figure 5). The bioluminescent signals were weaker in three of the six mice treated with compound 62520 than in the PBS-treated mice at 3 h post-infection. Bacterial clearance was observed in five out of six mice treated with compound 62520 at 24 h post-infection. These five mice survived at 7 days post-infection.

## 3. Discussion

In previous research, we screened small molecules inhibiting the *ompA* promoter activity of *A. baumannii* using reporter strain OH102 [29]. Several synthetic compounds, including compound 62520, were shown to inhibit *ompA* expression and attenuate biofilm formation in *A. baumannii* ATCC 17978. However, the plasmid carriage of gene constructs in reporter strain OH102 may affect the *ompA* promoter activity, due to the plasmid copy number and different location of the *ompA* promoter. Therefore, in this study, we constructed a new reporter strain, OH101—carrying a single copy *ompA* promoter and *ermAM* fusion in the chromosome—and then confirmed whether compound 62520 inhibited the *ompA* promoter activity in it under erythromycin conditions (30 μg/mL). Moreover, 95 structural derivatives of compound 62520 were screened using reporter strain OH101 in order to detect more active compounds that inhibit the *ompA* promoter activity. Of the tested chemicals, compound 62520 was the most active inhibiting the OH101 strain growth. It was also able to inhibit *ompA* expression and to attenuate biofilm formation in *A. baumannii* ATCC 17978 at sub-inhibitory concentrations (0.35–2.8 μg/mL). Next, we determined whether compound 62520 was also active in the inhibition of *ompA* expression and subsequent attenuation of biofilm formation in global epidemic CRAB isolates. It was revealed that compound 62520 inhibited *ompA* expression in most clinical CRAB isolates at sub-inhibitory concentrations, and *ompA* expression was highly correlated with biofilm formation in CRAB isolates, suggesting that compound 62520 inhibits OmpA-mediated biofilm formation in these isolates. However, *ompA* expression was not inhibited in the *A. nosocomialis* strain. Our results suggest that compound 62520 inhibits *ompA* expression in *A. baumannii* strains, subsequently attenuating biofilm formation at sub-inhibitory concentrations.

We previously showed that compound 62520 inhibited the growth of WT *A. baumannii* ATCC 17978 and this compound exhibited more than a 10-fold difference in the IC_50_ between the WT strain (7.95 μg/mL) and reporter strain OH102 (0.45 μg/mL) [29]. This result suggests that compound 62520 does not belong to classical anti-virulence agents, although it inhibits OmpA-mediated biofilm formation in *A. baumannii* strains at sub-inhibitory concentrations. Thus, its antimicrobial activity against *A. baumannii* strains was assessed in the present study. Compound 62520 exhibited antimicrobial activity against global epidemic CC208 CRAB isolates, as well as against two *A. baumannii* ATCC strains. The dose-responsiveness of the compound to bacterial growth was similar between *A. baumannii* ATCC 17978 and CRAB isolates. Moreover, no significant differences in its IC_50_ were observed between CRAB STs. These results suggest that the susceptibility of CRAB isolates to different classes of antimicrobials does not affect the antimicrobial activity of compound 62520. Compound 62520 also showed a bacteriostatic activity against the *A. nosocomialis* type strain, although it did not inhibit its *ompA* expression and biofilm formation. The antimicrobial activity was further tested against ESKAPE pathogens, except for *Klebsiella pneumoniae*. It was found that compound 62520 inhibited the growth of *E. coli* and *P. aeruginosa* ATCC strains but was less active in inhibiting the growth of *P. aeruginosa* than that of *Acinetobacter* strains. This compound showed a stronger effect on the growth inhibition of gram (+) bacteria— including *S. aureus* and *E. faecalis*— than gram (−) bacteria.

Building upon the in vitro inhibitory activities of compound 62520, the in vivo therapeutic effects were evaluated in mice infected with *A. baumannii* strains. In the in vivo imaging assay, no bioluminescence was observed in five out of six mice treated with compound 62520 at 24 h post-infection. However, one mouse infected with *A. baumannii* died, as the compound did not inhibit the intraperitoneal spread of bacteria in time. The treatment significantly increased the survival rates of both immunocompetent and neutropenic mice infected with *A. baumannii* ATCC 17978, and also those of immunocompetent mice infected with CRAB isolate. Neutrophils are important effector cells responsible for innate immunity against extracellular microbes, which may explain the increased survival rates of the *A. baumannii*-infected immunocompetent mice (50.0%), compared to those of the *A. baumannii*-infected neutropenic mice (23.5%). Compound 62520 showed a stronger effect on the survival of the latter (84.6%) than that of the former (76.9%), although the infectious doses were different between the two groups. In neutropenic mice treated with compound 62520, monocytes, macrophages, dendritic cells, and other immune effector cells in association with soluble effector molecules of innate immunity may be responsible for the elimination of *A. baumannii*. OmpA is directly or indirectly responsible for the pathogenic traits of *A. baumannii*, which include adherence to host cells [30], host cell death [31,32], resistance to complement-mediated bacterial killing [33], induction of pro-inflammatory responses [34], and invasiveness into blood vessels [24]. Moreover, OmpA expression is highly associated with disease progression and clinical outcomes in *A. baumannii* infections [35]. Both the inhibition of *ompA* expression at sub-inhibitory concentrations, and the bacteriostatic activity of compound 62520 may explain the increased survival rates of *A. baumannii*-infected mice. Our results suggest that this compound possesses a therapeutic potential against drug-resistant *A. baumannii* strains in vivo.

Compound 62520 inhibited *ompA* expression in *A. baumannii* strains, but not in *A. nosocomialis*, and was more active in inhibiting the growth of gram (+) bacteria (*S. aureus* and *E. faecalis*) than gram (−) bacteria (*E. coli*, *P. aeruginosa,* and *Acinetobacter*). These findings suggest that this compound specifically controls *ompA* expression in *A. baumannii* via either direct binding to the *ompA* promoter or binding to other molecules that regulate the transcription of *ompA* at sub-inhibitory concentrations. It is possible that compound 62520 controls bacterial growth by regulating molecules involved in the growth or metabolic pathways of ESKAPE pathogens at inhibitory concentrations. The underlying inhibitory mechanisms of compound 62520—specifically those regarding the bacteriostatic activity against ESKAPE pathogens and the down-regulation of *ompA* expression in *A. baumannii*—were not characterized in the present study and should be determined in future investigations.

In conclusion, the new small molecule compound 62520 was found to attenuate the OmpA-mediated biofilm formation through the transcriptional inhibition of *ompA* at sub-inhibitory concentrations and to exhibit bacteriostatic activity against global epidemic CRAB strains in vitro. This compound decreased the mortality of both immunocompetent and neutropenic mice infected with *A. baumannii* strains. Thus, compound 62520 represents a promising stepping stone to develop a novel therapeutic agent against drug-resistant *A. baumannii* infections.

## 4. Materials and Methods

### 4.1. Bacterial Strains, Plasmids, and Culture Conditions

The bacterial strains and plasmids used for the construction of the reporter strain are listed in Supplementary Table S2. *A. baumannii* ATCC 17978, *A. baumannii* ATCC 19606, *A. nosocomialis* ATCC 17903, *E. coli* ATCC 25922, *P. aeruginosa* ATCC 27853, *S. aureus* ATCC 29213, and *E. faecalis* ATCC 29212 were obtained from the American Type Culture Collection (ATCC). Eight ST191 CRAB isolates used to test the inhibition of *ompA* expression and biofilm formation by compound 62520 were selected based on the pulsotypes and expression levels of biofilm-associated genes in our previous study [36]. Clinical CRAB isolates were obtained from Kyungpook National University Hospital Culture Collection for Pathogens in Daegu, South Korea. Bacteria were grown in lysogeny broth (LB) or blood agar plates at 37 °C.

### 4.2. Chemical Compounds and Reagents

The chemical compounds used in this study were kindly provided by the Korea Chemical Bank (http://www.chembank.org/, accessed on 26 February 2016). Virstatin (4-[N-(1, 8-naphthalimide)]-n-butyric acid) was purchased from Enzo Life Science (Farmingdale, NY, USA). Compound 62520 was synthesized at the Korea Research Institute of Chemical Technology (KRICT) [37].

### 4.3. Screening of Chemical Compounds Inhibiting the Growth of Reporter Strain OH101

Ninety-five chemical compounds were selected based on their structural similarity with compound 62520 from 340,000 small molecules available from the Korea Chemical Bank. Reporter strain OH101 was cultured in LB for 20 h and then the cultured bacteria were inoculated into Mueller-Hinton (MH) broth supplemented with erythromycin (30 μg/mL) to obtain a final population to be transferred to 96-well plates at 6.0 × 10^6^ CFUs/well [29]. The chemical compounds (2.5 μM) were added to each well. *A. baumannii* ATCC 17978 was cultured with the compounds in MH broth without erythromycin to assess the growth inhibition of the WT strain. The same volume of dimethyl sulfoxide (DMSO)—used to dissolve the chemical compounds—was added into the wells as a control. The plates were incubated for 24 h and the growth of bacteria was measured using a microplate reader (Molecular Devices, Sunnyvale, CA, USA) with optical density set at 600 nm (OD_600_). To determine the growth inhibition of reporter strain OH101 by chemical compounds, the following equation was used: OD_600_ at 24 h − OD_600_ at 0 h in the presence of chemical compounds/OD_600_ at 24 h − OD_600_ at 0 h in the absence of chemical compounds [29].

### 4.4. Sodium Dodecyl Sulfate-Polyacrylamide Gel Electrophoresis and Western Blotting

*A. baumannii* ATCC 17978 was grown in LB containing compound 62520 with shaking for 24 h. Bacterial cells were harvested and then disrupted by sonication (Branson Ultrasonics Corp., Danbury, CT, USA). The samples were centrifuged to remove bacterial debris and the supernatant was harvested by ultracentrifugation at 100,000× *g* for 1 h at 4 °C. The pellet was resuspended in 10 mM HEPES buffer with 2% sodium lauryl sarcosine (Sigma-Aldrich, St. Louis, MO, USA) and was incubated for 30 min at room temperature. The samples were then centrifuged at 100,000× *g* for 1 h at 4 °C. The pellet containing outer membrane fractions was resuspended in a small volume of PBS. The outer membrane proteins (10 μg) were resuspended in the sample buffer used for the sodium dodecyl sulfate-polyacrylamide gel electrophoresis (SDS-PAGE) and were boiled for 10 min to obtain denaturation. The proteins were separated on a 12% SDS-PAGE gel and stained with Coomassie brilliant blue R-250 (Bio-Rad, Hercules, CA, USA); they were then electroblotted onto nitrocellulose membranes to perform western blotting. The membranes were first incubated with a polyclonal anti-rabbit OmpA immune serum [38], and then with a secondary antibody coupled to horseradish peroxidase. Finally, they were developed using an enhanced chemiluminescence system (Amersham Pharmacia Biotech, Piscataway, NJ, USA).

### 4.5. Quantitative Real-Time PCR

The expression of *ompA* was determined through qPCR. Bacteria were cultured in MH broth containing compound 62520 for 24 h. Total RNA was obtained using the Qiagen RNeasy mini kit (Qiagen, Hilden, Germany), according to the manufacturer’s instructions. Reverse transcription was performed to synthesize cDNA using 1.5 μg of total RNA, random hexamer primers, and M-MLV reverse transcriptase (Thermo Scientific, Waltham, MA, USA). The specific primers for the *ompA* and 16S rRNA genes were: 5′-TTG CAC TTG CTA CTA TGC TTG TTG-3′ and 5′-TGG CTG TCT TGG AAA GTG TAA CC-3′ for *ompA*, and 5′-ACT CCT ACG GGA GGC AGC AGT-3′ and 5′-TAT TAC CGC GGC TGC TGG C-3′ for 16S rRNA. The quantification of gene transcripts was performed using the TOPreal™ qPCR 2X PreMIX (SYBR Green with high ROX) (Enzynomics) on an ABI PRISM 7500 Real-Time System (Applied Biosystems, Foster City, CA, USA), according to the manufacturer’s instructions. Fold changes in gene expression were calculated using the comparative C*t* method and samples were normalized to 16S rRNA expression. The assays were performed in three independent experiments.

### 4.6. Biofilm Formation Assay

The overnight cultures of bacteria, grown in LB without sodium chloride, were diluted 100-fold with the same medium, and then compound 62520 was added to the 200 μL of bacterial suspension in a 96-well plate. Two wells were used for each bacterial strain. Bacteria were incubated for 24 h at 30 °C under static conditions. Total bacterial growth (OD_600_) was measured using the microplate reader. Planktonic bacteria were removed and the plate was washed twice with 200 μL of PBS. Biofilm cells were stained with 0.1% (*w*/*v*) crystal violet solution for 15 min. The biofilms were quantified at OD_570_ using a biofilm cell-associated dye and were normalized to bacterial growth (OD_600_). The biofilm assay was performed in triplicate in three independent experiments.

### 4.7. Bacterial Growth Inhibition Analyses

The 6.0 × 10^6^ CFUs of bacteria suspended in MH broth were inoculated into each well of the 96-well plate and then compound 62520 were added at concentrations ranging from 1 μg/mL to 32 μg/mL or 512 μg/mL. The same DMSO volume used to dissolve the compound was added as a control. Bacteria were incubated for 20 h at 37 °C under static conditions and bacterial growth was measured using the microplate reader at OD_600_. Bacterial growth analyses were performed in two independent experiments. The MIC was determined by the lowest concentration of compound 62520 that inhibited the visible growth of bacteria.

### 4.8. Bioluminescence Imaging of Mice Infected with A. baumannii

Eight-week-old female BALB/c mice were maintained under specific pathogen-free conditions. Mice were intraperitoneally infected with 1.0 × 10^9^ CFUs of *A. baumannii* ATCC 17978-lux strain [24]. Subsequently, 2 h after bacterial infection, compound 62520 (2.8 mg/kg in 100 μL PBS) was intraperitoneally injected in the opposite side of their abdomen. The control mice were injected with the same volume of PBS. The infected mice were subjected to bioluminescence analysis using the IVIS Lumina XRMS system (Perkin Elmer, Waltham, MA, USA). Images were captured at the indicated time points (3 and 24 h post-infection), and luminescence as radiance (p/sec/cm^2^/sr) was acquired by a computer using Living Image Software (Perkin Elmer). All animal experiments were performed in accordance with regulations established by the Institutional Animal Care and Use Committee of Kyungpook National University.

### 4.9. Survival of A. baumannii-Infected Mice Treated with Compound 62520

In order to evaluate the effect of compound 62520 on survival, immunocompetent and neutropenic eight-week-old female BALB/c mice were intraperitoneally infected with 1.0 × 10^9^ CFUs and 5 × 10^8^ CFUs of *A. baumannii* ATCC 17978, respectively, and, 2 h after bacterial injection, they were intraperitoneally injected with compound 62520 (100 μL of injection volume) in the opposite side of the abdomen. Neutropenia had been induced by intraperitoneal injections of cyclophosphamide (150 mg/kg) 4 and 1 day prior to the bacterial injection. The survival of mice was evaluated for 7 days after bacterial injection. To evaluate the effect of compound 62520 on clinical *A. baumannii* isolates, immunocompetent mice were intraperitoneally infected with 2.0 × 10^8^ CFUs of *A. baumannii* 2140, and were then intraperitoneally injected with compound 62520 (5.6 mg/kg in 100 μL of PBS) 2 h after bacterial injection. All control mice were injected with the same volume of PBS.

### 4.10. Statistics

Statistical analyses were carried out using GraphPad Prism 5.0 software (San Diego, CA, USA). One-way analysis of variance with Dunnett’s post-hoc analysis and Student’s *t*-tests were performed to compare control and experimental groups. Kaplan-Meier curve analysis and log-rank test were conducted to assess the survival rate of mice treated with compound 62520. Differences at *p* < 0.05 were considered statistically significant.

## Figures and Tables

**Table 1:** Antibacterial activity of compound 62520 against clinical carbapenem-resistant *A. baumannii* isolates.

Sequence Type	MIC (μg/mL)	IC_50_ (μg/mL) *
ST191 (*n* = 5)	>32	8.62 ± 3.46
ST208 (*n* = 5)	>32	7.23 ± 2.41
ST369 (*n* = 5)	>32	6.58 ± 2.63
ST451 (*n* = 5)	>32	8.30 ± 2.08
ST784 (*n* = 5)	>32	6.35 ± 3.05

* The half-maximal inhibitory concentration of compounds (IC_50_) was the compound 62520 concentration showing 50% of the maximal inhibition. This was calculated as follows: relative maximal inhibition% − 50% × (relative maximal inhibition% − relative minimal inhibition%).

## Data Availability

The authors confirm that the data supporting the findings of this study are available within the article and its Supplementary Materials.

## Supplementary Materials

The following are available online at https://www.mdpi.com/article/10.3390/ijms222212257/s1.
