# Supplementary material for: Therapeutic Effects of Inhibitor of ompA Expression against Carbapenem-Resistant Acinetobacter baumannii Strains
Source: Int J Mol Sci. 2021 Nov 12;22(22):12257. doi: 10.3390/ijms222212257 (PMC8623844; doi:10.3390/ijms222212257)
Supplement: Supplementary file 1 [file ijms-22-12257-s001.zip › Supplementary Figures.pptx]

## Slide 1
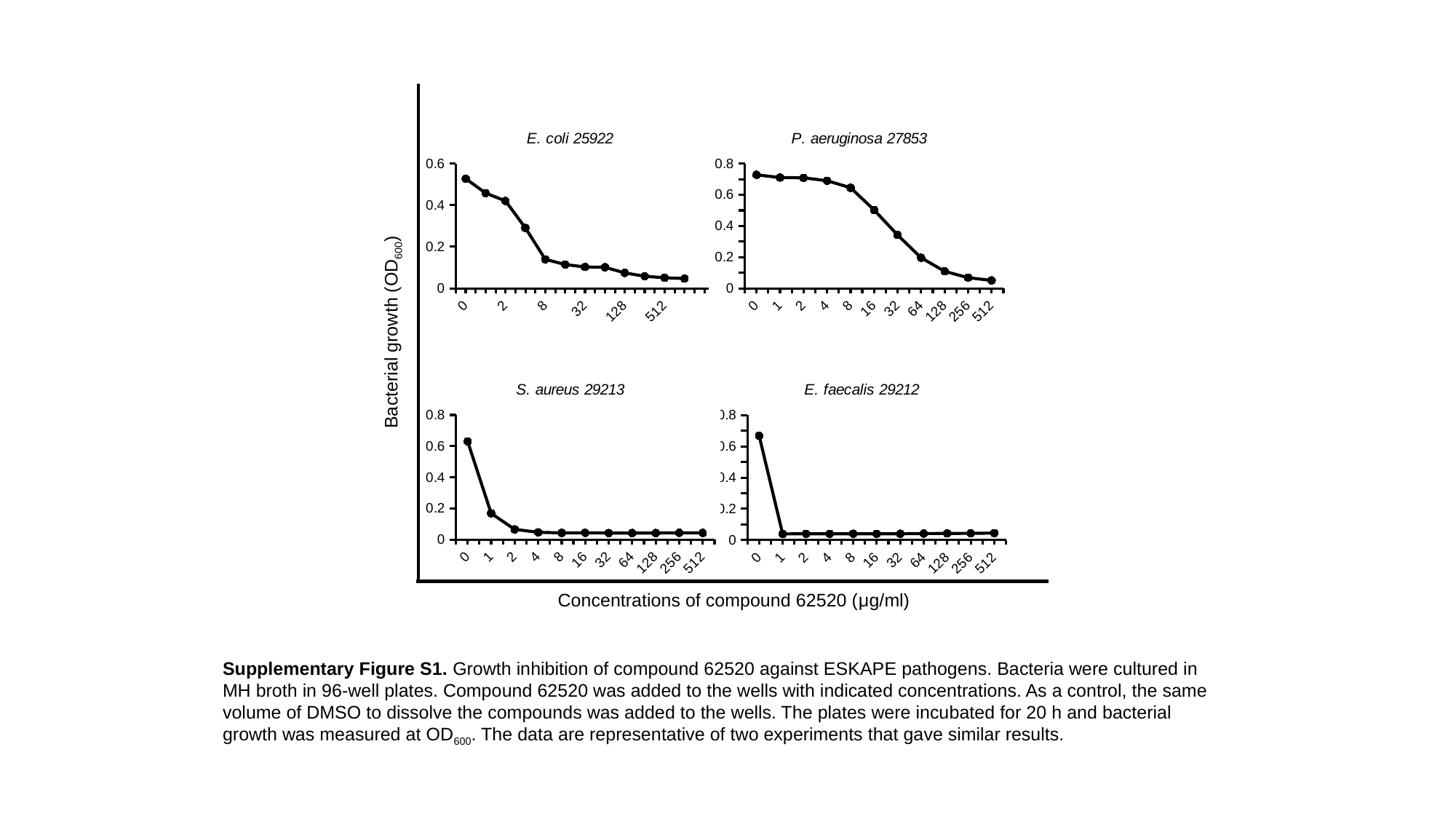

Bacterial growth (OD600)
### Chart: E. coli 25922
| Category | E.coli 25922 |
|---|---|
| 0 | 0.5275 |
| 1 | 0.4583 |
| 2 | 0.4205 |
| 4 | 0.2903 |
| 8 | 0.1394 |
| 16 | 0.1145 |
| 32 | 0.1026 |
| 64 | 0.1013 |
| 128 | 0.0741 |
| 256 | 0.0579 |
| 512 | 0.0507 |
### Chart: P. aeruginosa 27853
| Category | P.aerusinosa 27853 |
|---|---|
| 0 | 0.7283 |
| 1 | 0.7106 |
| 2 | 0.7088 |
| 4 | 0.6902 |
| 8 | 0.6452 |
| 16 | 0.5029 |
| 32 | 0.3421 |
| 64 | 0.1962 |
| 128 | 0.1091 |
| 256 | 0.0682 |
| 512 | 0.0508 |
### Chart: S. aureus 29213
| Category | S.aureus 29213 |
|---|---|
| 0 | 0.6309 |
| 1 | 0.1689 |
| 2 | 0.0663 |
| 4 | 0.0475 |
| 8 | 0.0438 |
| 16 | 0.0442 |
| 32 | 0.0429 |
| 64 | 0.0428 |
| 128 | 0.0429 |
| 256 | 0.0443 |
| 512 | 0.0437 |
### Chart: E. faecalis 29212
| Category | E.faecalis 29212 |
|---|---|
| 0 | 0.6685 |
| 1 | 0.0391 |
| 2 | 0.0399 |
| 4 | 0.0397 |
| 8 | 0.0401 |
| 16 | 0.0398 |
| 32 | 0.0401 |
| 64 | 0.0411 |
| 128 | 0.042 |
| 256 | 0.0428 |
| 512 | 0.0443 |Concentrations of compound 62520 (μg/ml)
Supplementary Figure S1. Growth inhibition of compound 62520 against ESKAPE pathogens. Bacteria were cultured in MH broth in 96-well plates. Compound 62520 was added to the wells with indicated concentrations. As a control, the same volume of DMSO to dissolve the compounds was added to the wells. The plates were incubated for 20 h and bacterial growth was measured at OD600. The data are representative of two experiments that gave similar results.

## Slide 2
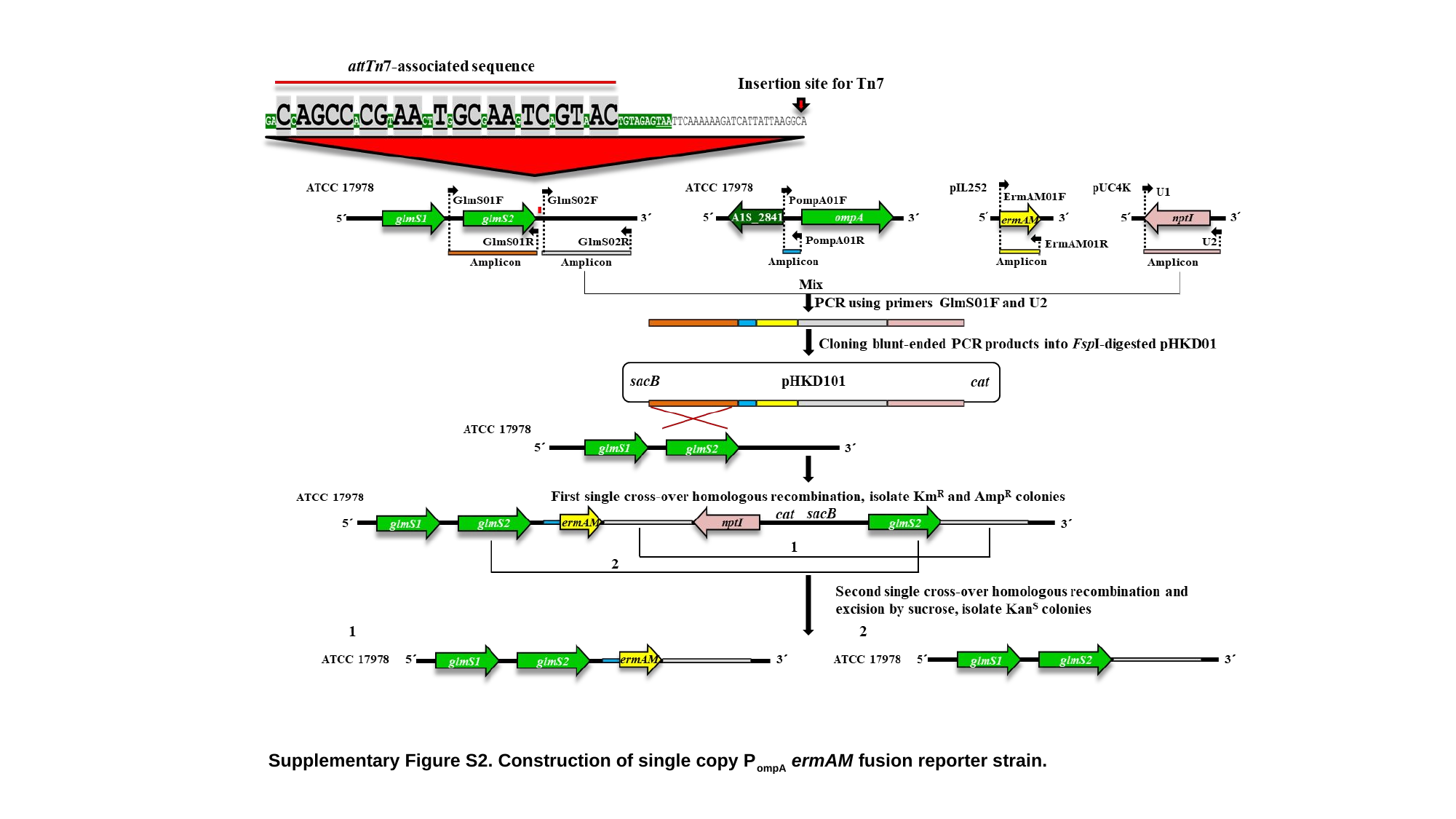

Supplementary Figure S2. Construction of single copy PompA ermAM fusion reporter strain.
