# Supplementary material for: Therapeutic Effects of Inhibitor of ompA Expression against Carbapenem-Resistant Acinetobacter baumannii Strains
Source: Int J Mol Sci. 2021 Nov 12;22(22):12257. doi: 10.3390/ijms222212257 (PMC8623844; doi:10.3390/ijms222212257)
Supplement: Supplementary file 1 [file ijms-22-12257-s001.zip › Supplementary Tables and Materials and Methods.pdf]

Supplementary Table S1. Screening of compound 62520 derivatives using reporter strain OH101.

No.	Compound ID	Chemical structure	Molecular weight	% inhibition at 2.5 μM[a]
1	62520	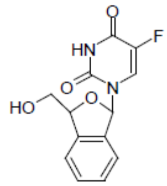	278.24	72.15
2	4681	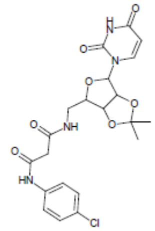	478.89	30.44
3	136158	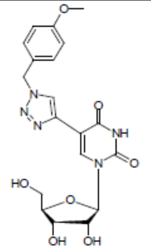	431.41	28.80
4	59118	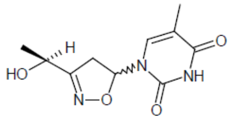	239.23	28.26
5	63979	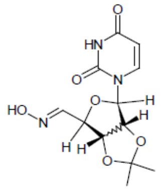	297.27	27.53
6	203112	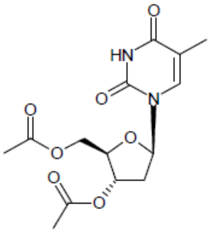	326.31	25.65
7	136160	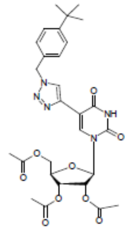	583.60	24.98

8	202044	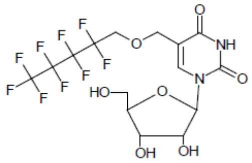	506.28	24.65
9	203049	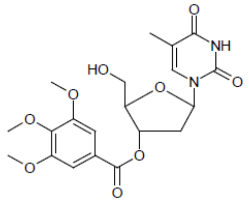	436.42	24.47
10	62333	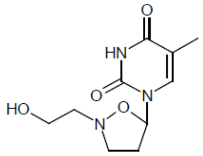	241.25	23.39
11	136155	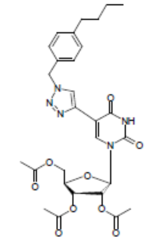	583.60	23.01
12	62389	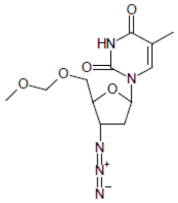	311.30	22.77
13	64097	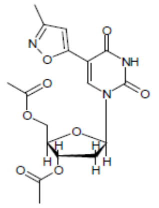	393.36	22.77
14	201824	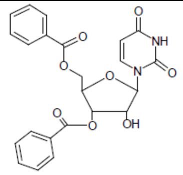	452.42	21.40
15	30625	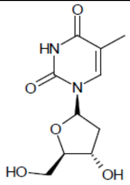	242.23	20.97

[a] The following equation was used to determine the % inhibition: OD_{600} at 24 h - OD_{600} at 0 h in the presence of chemical compounds/OD_{600} at 24 h - OD_{600} at 0 h in the absence of chemical compounds.

Supplementary Table S2. Bacterial strains and plasmids used for the construction of reporter strain OH101.

Bacteria or plasmids	Relevant characteristics[a]	Reference or source
Bacteria		
A. baumannii		
ATCC 17978	Wild-type strain	ATCC
OH101	ATCC 17978 with *ermAM* under control of *ompA* promoter	This study
E. coli		
S17-l λ pir	λ-pir lysogen; thi pro hsdR hsdM+ recA RP4-2 Tc::Mu-Km::Tn7;Tpr Smr; host for π-requiring plasmids; conjugal donor	[1]
Plasmids		
pUC4K	pUC4 with *nptI*; Amp^r, Km^r	Pharmacia
pIL252	Erm^r, oripAMβ1	[2]
pHKD01	Suicide vector; *ori*R6K, *sacB*, Cm^r	[3]
pOH101	pHKD01 with *ermAM* coding region under control of *ompA* promoter of *A. baumannii* ATCC17978	This study

[a] Amp^r, ampicillin-resistant; Cm^r, chloramphenicol-resistant; Erm^r, erythromycin-resistant; Km^r, kanamycin-resistant.

Supplementary Table S3. Oligonucleotides used in this study.

Primers[a]	Sequence (5′→3′)[b]	Use
PompA01F	ATT CAA AAA AGA TCA TTA TTA AGG CAA TCA CAA GAA TTA CGA GTG TTA TAG TGA	Amplification of the *ompA* promoter region
PompA01R	GAG AAT ATT TTA TAT TTT TGT TCA TAG TAG CAA GTG CAA TAC GAC TCA AT	
ErmAM01F	ATG AAC AAA AAT ATA AAA TAT TCT CAA AAC TT	Amplification of the *ermAM* coding region
ErmAM01R	TCA ATC AAT TAT TAA AGC AGG TGA TGA CCT CTT TAG CTC CTT GGA AGC	
GlmS01F	TGG TTT GAG CAA TTG ACT TGG	Amplification of the *glmS* upstream
GlmS01R	GCC TTA ATA ATG ATC TTT TTT GAA TTA CT	
GlmS02F	ATC ACC TGC TTT AAT AAT TGA TTG ATT A	Amplification of the *glmS* downstream
GlmS02R	GCA ACA CCT TCT TCA CGA GGC AGA CAG TCG GTT TTA GCA GAC CGT AC	
U1	GTC TGC CTC GTG AAG AAG GTG	Amplification of the kanamycin-resistance cassette
U2	GAT CCG TCG ACC TGC AGG	

[a] The primers were designed using the genome sequences of *A. baumannii* ATCC 17978 (GenBank accession number NC009085).

[b] Regions of oligonucleotides that are not complementary to the corresponding templates are underlined.

Supplementary Materials and Methods

Construction of the reporter strain

The reporter strain carrying single copy of the *ompA* promoter and *ermAM* fusion in the chromosome was constructed for the bacterial growth-based assay system to screen small molecules that inhibit the *ompA* promoter activity (Supplementary Figure S3). The *ermAM* coding regions under the control of the *ompA* promoter were inserted into an *attTn*7 site located downstream of the *glmS* [4] encoding glucosamine-fructose-6-phosphate aminotransferase by a modified markerless gene deletion method [3]. The *ompA* promoter region and the upstream and downstream *glmS* regions were amplified from the genomic DNA of *A. baumannii* ATCC 17978 using the primer pairs PompA01F/PompA01R, GlmS01F/GlmS01R, and GlmS02F/GlmS02R, respectively (Supplementary Table S3). The *ermAM* coding region and a kanamycin-resistance cassette were amplified using the primer pairs ErmAM01F/ErmAM01R and U1/U2 (Supplementary Table S3), respectively. The pIL252 and pUC4K plasmids were used as templates for the *ermAM* coding region and kanamycin-resistance cassette, respectively. In particular, the PompA01F and PompA01R primers contained 25 additional nucleotides at the 5′ end, and were homologous to the upstream *glmS* region and *ermAM* coding region, respectively. Similarly, the ErmAM01R and GlmS02R primers carried 25 additional nucleotides at the 5′ end, and were homologous to the downstream *glmS* region and kanamycin-resistant cassette, respectively. The five amplicons obtained in the first PCR were mixed at equimolar concentrations and were subjected to overlap extension PCR with GlmS01F and U2 primers. The combined DNA fragment was then cloned into *Fsp*I-digested pHKD01 to yield pOH101 (Supplementary Table S2). The plasmid was integrated into the chromosome of *A. baumannii* ATCC 17978 using conjugation-based gene transfer and homologous recombination. In the first single cross-over homologous recombination, merodiploids that possessed an integrated copy of pHKD01 carrying the recombinant DNA were obtained on LB

agar plates containing kanamycin (50 μg/ml) and ampicillin (100 μg/ml). A second single cross-over homologous recombination in the bacteria was achieved using *sacB*, which conferred sucrose sensitivity, on LB agar plates containing sucrose. The bacteria that were sensitive to kanamycin were selected, and the target gene insertion was then confirmed by PCR analysis. The exact insertion of *ermAM* into the *ompA* site and the deletion of ORF of *ompA* in the chromosome of *A. baumannii* ATCC 17978 were also confirmed by PCR analysis.

References

1. Clifford, R.J.; Milillo, M.; Prestwood, J.; Quintero, R.; Zurawski, D.V.; Kwak, Y.I.; Waterman, P.E.; Lesho, E.P.; Gann, P.M. Detection of bacterial 16S rRNA and identification of four clinically important bacteria by real-time PCR. *PLoS ONE.* **2012,** 7, e48558.
2. Simon, D.; Chopin, A. Construction of a vector plasmid family and its use for molecular cloning in *Streptococcus lactis*. *Biochimie.* **1988,** 70, 559-566.
3. Oh, M.H.; Lee, J.C.; Kim, J.; Choi, C.H.; Han, K. Simple method for markerless gene deletion in multidrug-resistant *Acinetobacter baumannii*. *Appl. Environ. Microbiol.* **2015,** 81, 3357-3368.
4. Kumar, A.; Dalton, C.; Cortez-Cordova, J.; Schweizer, H.P. Mini-Tn7 vectors as genetic tools for single copy gene cloning in *Acinetobacter baumannii*. *J. Microbiol. Methods.* **2010,** 82, 296-300.
